# A comparison of linkage to HIV care after provider-initiated HIV testing and counselling (PITC) versus voluntary HIV counselling and testing (VCT) for patients with sexually transmitted infections in Cape Town, South Africa

**DOI:** 10.1186/1472-6963-14-350

**Published:** 2014-08-18

**Authors:** Natalie Leon, Catherine Mathews, Simon Lewin, Meg Osler, Andrew Boulle, Carl Lombard

**Affiliations:** Health Systems Research Unit (HSRU), Medical Research Council of South Africa (MRC), P.O. Box 19070, Tygerberg, 7507 Cape Town, Western Cape Province Republic of South Africa; School of Public Health and Family Medicine, University of Cape Town (UCT), Cape Town, Western Cape Province Republic of South Africa; Global Health Unit, Norwegian Knowledge Centre for the Health Services, Oslo, Norway; Centre for Infectious Disease Epidemiology and Research (CIDER), UCT, Cape Town, Western Cape Province Republic of South Africa; Provincial Government of the Western Cape (PGWC), Cape Town, Western Cape Province Republic of South Africa; Biostatistics Unit, MRC, Cape Town, Western Cape Province Republic of South Africa

**Keywords:** Routine HIV screening, Linkage to HIV care, Sexually transmitted infections, Controlled trial, South Africa

## Abstract

**Background:**

We examined linkage to care for patients with sexually transmitted infection who were diagnosed HIV-positive via the provider-initiated HIV testing and counselling (PITC) approach, as compared to the voluntary counselling and testing (VCT) approach, as little is known about the impact of expanded testing strategies on linkage to care.

**Methods:**

In a controlled trial on PITC (Cape Town, 2007), we compared HIV follow-up care for a nested cohort of 930 HIV-positive patients. We cross-referenced HIV testing and laboratory records to determine access to CD4 and viral load testing as primary outcomes. Secondary outcomes were HIV immune status and time taken to be linked to HIV care. Logistic regression was performed to analyse the difference between arms.

**Results:**

There was no difference in the main outcomes of patients with a record of CD4 testing (69.9% in the intervention, 65.2% in control sites, OR 0.82 (CI: 0.44-1.51; p = 0.526) and viral load testing (14.9% intervention versus 10.9% control arm; OR 0.69 (CI: 0.42-1.12; p = 0.131). In the intervention arm, ART-eligible patients (based on low CD4 test result), accessed viral load testing approximately 2.5 months sooner than those in the control arm (214 days vs. 288 days, HR: 0.417, 95% CI: 0.221-0.784; p = 0.007).

**Conclusion:**

The PITC intervention did not improve linkage to CD4 testing, but shortened the time to viral load testing for ART-eligible patients. Major gaps found in follow-up care across both arms, indicate the need for more effective linkage-to-HIV care strategies.

**Trial registration:**

Current Controlled Trials ISRCTN93692532

**Electronic supplementary material:**

The online version of this article (doi:10.1186/1472-6963-14-350) contains supplementary material, which is available to authorized users.

## Background

The ultimate aim of approaches to expand HIV testing is to improve access to prevention, care and treatment services, through early detection of HIV [1]. The secondary prevention benefits of early detection and antiretroviral treatment (ART) has been demonstrated by studies using the ‘test and treat’ strategy - aimed at reducing HIV transmission as well as morbidity and mortality [2, 3]. Provider-initiated HIV testing and counselling (PITC) is an approach to expand HIV testing through integrating the routine offer of HIV testing into standard care in medical settings [1, 4]. The focus of PITC studies has been largely on feasibility, acceptability and increasing test uptake and there have been positive findings in all three areas [5–8]. Questions remain about the ethics of increasing the number of patients who receive a HIV diagnosis when many may not be able to access the survival gains associated with ART, especially in low-resource settings [9–13]. Despite the aim of improved access to care and the ethical concerns, little is known of the impact of PITC on linkage to care, as compared to other approaches such as voluntary counselling and testing (VCT).

Claims that PITC may be able to increase access to earlier diagnosis and care compared to other testing approaches (through, for instance, integrating HIV testing into standard clinical care), are still unsubstantiated [10, 13]. The little evidence available does not appear to bear out the concerns about reduced access for patients diagnosed HIV-positive via the PITC approach. A randomised controlled study of PITC for TB patients in South Africa found no difference in linkage to care compared to VCT with respect to referrals for HIV medical care or prescriptions for cotrimoxazole prophylaxis [14]. Similarly, a review of routine data for PMTCT patients after the introduction of routine HIV screening in Botswana in 2004, found that significantly more women knew their HIV status at delivery, but that there was no difference to the proportions who had been initiated on antiretroviral therapy [15]. Few studies have pointed to a positive impact of PITC on linkage to care as compared to standard opt-in approaches. A Zimbabwe study in 2005 compared mother-to-child transmission (PMTCT) service indicators during a 6 month routine HIV testing period with the prior opt-in testing period and found significantly more women in the routine testing period received their test results and were post-test counselled and more mother-infant pairs were seen at their 6 week follow-up visits [16]. Stronger evidence emerged from a South African retrospective review that found a statistically significant temporal association between the introduction of PITC for patients with TB in 2005, and subsequent increased referrals to ART (from 16% in the period 2002–2005 to 34.7% in 2007–2008) [17].

Evidence on linkage to care in relation to different HIV testing strategies is often variable and difficult to interpret as definitions of linkage to care are not uniform across studies [18]. The variety of HIV testing strategies, patient populations and health system contexts that are studied also make it difficult to compare findings on linkage to care. For instance, two South African retrospective studies had contradictory findings about whether patients who accessed routine, provider-initiated HIV testing (as opposed to self-initiated testing), had better linkage to care – and it was not clear to what extend the testing strategies in the two studies are comparable. The one study examined the PHC records of a random sample of HIV positive patients living in one geographical community in Cape Town, selecting from antenatal, STI and TB patients as well as those who self-initiated VCT. The authors suggested that patients who tested via routine screening (such as for antenatal patients) had better linkage to HIV care than those who self-initiated [19]. The other study examined linkage to care for an out-patient cohort of HIV positive patients (excluding antenatal patients) at a semi-private and a state-aided hospital and suggested that those who self-initiated HIV testing may be more motivated to seek follow-up care than those who were medically referred [20]. The variation in PITC strategies used (often with insufficient description of the intervention), makes it harder to interpret findings and compare results across PITC studies. There may not be one single answer to how PITC impacts on linkage to care and part of the variability may be due to the contextual variables mentioned above.

The cascade of care for HIV positive patients comprises various stages. The first is from HIV testing to staging for HIV immune status through CD4 testing and clinical examination (often referred to as ‘linkage to care’), next is the period between the staging and becoming ART-eligible, followed by initiation on ART, and finally the stage from ART initiation to retention in care [2, 18]. In this study, the focus is on the first two stages of care, that is, the stage of linkage to CD4 testing (and determining ART eligibility) and the stage of linkage to viral load testing (the latter as a proxy for initiation on ART).

A systematic review of linkage to care between HIV testing and ART initiation in Sub Saharan Africa showed that by the end of 2009, only about a third (33%) of those HIV positive patients who needed ART (based on their low CD4 count, also referred to as ‘ART-eligible’), were receiving treatment [18]. With the gaps in linkage to care, especially prior to ART initiation [18, 20, 21], it remains important to continue evaluating the effect of PITC on HIV follow-up care with different patient groups, especially in high HIV prevalence and low-resource settings such as South Africa. Moreover, given the strong association between sexually transmitted infection (STI) and the risk of acquiring and transmitting HIV [22, 23], HIV prevention, HIV detection and linkage to HIV care amongst STI patients are of particular importance; and there is a gap in evidence on the effect of expanding HIV testing strategies, such as PITC on linkage to care for this patient group.

Clinical guidelines for linkage to care in South Africa required CD4 testing and clinical examination for all newly diagnosed HIV-positive patients as soon as possible, usually within a week of testing. At the time of the study, patients not yet eligible for ART (CD4 > 200 cells/mm^3^ and no clinical symptoms of AIDS) would be advised to attend every 6 months for a clinical assessment and CD4 count monitoring. If ART-eligible (CD4 ≤ 200 cells/mm^3^ and/or HIV clinical stage IV) patients would be referred for ART initiation. For patients referred for ART initiation, clinical guidelines required baseline viral load testing at the point of starting ART. (During the course of this study, this requirement was changed to viral load testing within 6–9 months of starting ART). Retention in care for ART was monitored by regular viral load testing at 6 monthly intervals. In this study, linkage to care refers to whether a patient accessed a CD4 test after testing HIV positive and whether they accessed a viral load test if they were eligible to be initiated on ART during the observation period. Access to viral load testing was used as a proxy for having been linked to ART.

The main non-randomised controlled trial, of which this study is nested within, found that participants in the PITC arm in public STI services in Cape Town, were more likely to accept HIV testing compared with those in the VCT arm [24]. The aim of this nested follow-up study was to compare linkage to care (as measured by CD4 and viral load testing) of STI patients diagnosed HIV-positive in clinics with PITC versus those diagnosed HIV-positive in clinics with the standard VCT approach. Secondary indicators were HIV immune status (CD4 count) and time taken to linkage to care.

## Methods

### Study setting

The study was performed in the publicly funded primary healthcare (PHC) services in Cape Town, South Africa. Although the Western Cape Province and Cape Town have amongst the lowest HIV prevalence in the country the prevalence varies dramatically between sub-districts within the city. For instance, in 2005, the HIV prevalence for pregnant women was estimated at 15.7% for Cape Town, whilst on a sub-district level the figures ranged from as low as 5.1% to as high as 32.6% [25]. The sub-districts with the highest figures also tended to be those with the poorest socio-economic profile.

### Study design

This nested cohort study was conducted in the context of a controlled trial (ISRCTN 93692532) that evaluated whether PITC increased HIV testing rates in 7 intervention and 14 control primary health care (PHC) clinics in Cape Town. Details of the trial study design, setting, the PITC intervention and results were described elsewhere [24]. This follow-up study reviewed retrospective laboratory records of CD4 and viral load tests of the 930 patients who were diagnosed HIV-positive during the period of the controlled trial between January 2007 and June 2007. Laboratory records of CD4 and viral load testing were used as indicators of linkage to care because these tests can only be requested by a nurse or doctor. Access to these tests was used as a proxy for whether patients received the appropriate type of care, though it is acknowledged that this is not a comprehensive indicator of linkage to care. The existence of a laboratory test record does not indicate that the patient returned and was informed of the test result and this is noted as a limitation in the Discussion section.

The main controlled trial was done in an operational setting which precluded randomisation of sites. In the main trial, the intervention and control clinics were compared at baseline on multiple demographic and service delivery factors (data not shown- see Additional file 1).

The primary outcomes were i) the proportion of HIV-positive patients who had had a CD4 test record and ii) of those with a CD4 test record, the proportion of ART-eligible patients with a viral load test record. Secondary outcomes were HIV immune status (as reflected by their CD4 count) and the time taken between HIV testing and CD4 testing and between CD4 testing and viral load testing. The study hypothesised that the PITC intervention would offer more opportunity for linkage to care compared to the VCT option (due to the closer, routine involvement of clinical staff in the HIV testing process).

### The PITC intervention and HIV linkage to care

STI services and HIV testing is available to all patients in PHC clinics, free of charge. HIV testing is usually provided by trained HIV lay counsellors via the VCT approach which require patients to self-initiate or be medically referred for HIV pre- and post-test counselling. In clinics with the PITC intervention, the STI nurses offered HIV testing routinely as part of their STI consultation, through a series of brief steps aimed at assessing test readiness, getting consent and performing the rapid test. After performing the HIV test, the nurse linked the patient with the HIV lay counsellor for the test result and post-test counselling.

The differences and similarities between the standard VCT approach and the adapted version of the PITC approach used in this study, is outlined in Table 1. In both intervention and control sites, the reason for the clinic visit is to seek treatment for STI. The difference in the PITC sites was that all STI patients would routinely be offered an HIV test as part of the STI consultation, whereas in the control sites, patients would not automatically be offered HIV testing in STI consultation. In the control sites, there was no standardised requirement for the clinician to raise the issue of HIV testing in the STI consultation, and in most cases, patients in control sites would only be referred to the HIV testing and counselling services, based on medical reasons (medical referral), such as the presence of HIV-related symptoms. Such patients would usually make a separate appointment for HIV testing and counselling with lay health counsellors, or they may choose to ignore the medical referral.

**Table 1:** **Similarities and differences between the VCT and the PITC interventions for patients seeking STI care in Cape Town**

	Voluntary counselling and testing for HIV for STI patients in control sites	Provider–initiated HIV testing and counselling for STI patients in intervention sites
**Patient access**	• Patients come to the clinic to seek care for an STI complaint.	• Patients come to the clinic to seek care for an STI complaint.
	• HIV testing is not offered by the STI nurse as part of the standard clinical care.	• The STI nurse routinely offers all STI patients an HIV test as part of the standard STI clinical care.
	• The STI nurse may refer some STI patients for HIV testing, usually for medical reasons.	• The STI patient is asked to opt-out of HIV testing during the STI consultation.
	• Should patient choose to adhere to the medical referral, then a separate clinic visit is usually required for the HIV counselling and testing to be done.	
**Providers**	• Usually provided by trained lay counsellors.	• Professional healthcare providers (STI nurses) trained to provide PITC.
	• Basic counselling training can be lengthy (10 to 20 days).	• Training is short (2 days) and is focused on how to offer the test and how to get informed consent from patients.
**Primary purpose of the intervention**	• The primary purpose is to promote uptake of HIV testing and to link people to HIV care and prevention services.	• The primary purpose is, similarly, to promote uptake of HIV testing and increase the number of people who know their HIV status.
	• The emphasis is on assessing patient readiness to test, and the counsellor is supposed to remain neutral about the choice (and not to promote taking the HIV test as the preferred option).	• The intervention also aims to integrate HIV testing efficiently into a regular STI consultation, while still respecting the need for patient informed consent.
		• The provider can promote HIV testing as the medically recommended option (rather than remaining neutral about the preferred choice).
**Pre-test encounter**	• Patient-centred counselling techniques focus on promoting an informed decision and include basic HIV information, risk assessment, an assessment of readiness to test, and risk reduction messages.	• Offer of HIV testing is introduced using regular clinical communication as part of the STI consultation. This involves a brief explanation of why an HIV test is recommended in the context of an STI consultation, a brief assessment of the patient’s readiness to test for HIV, offering the HIV test and opportunity for the patient to ask questions. Risk assessment and risk reduction are dealt with as part of the regular STI consultation.
	• Written informed consent for testing is obtained.	• Written informed consent for testing is obtained.
	• Can take up to 25 minutes.	• Intervention is meant to add maximum 5 to 10 minutes to the STI consultation when efficiently integrated.
**The HIV test**	• Due to limits to their scope of practice, lay counsellors cannot perform the rapid HIV tests themselves.	• The nurse does the HIV rapid test along with other blood tests during the STI consultation, which reduces waiting time for patients.
	• The rapid test is performed by a nurse, which may involve some waiting time.	
**Post-test and follow-up care**	• The nurse communicates the result of the rapid HIV test to the lay counsellor.	• The nurse refers the patient to a lay counsellor in the facility, to receive the HIV test result and post-test counselling.
	• The lay counsellor then informs the patient and provides post-test counselling.	• The patient may need to wait for a lay counsellor to be available.
	• The primary focus is on providing emotional support for HIV-positive patients and linking them to care, as well as providing risk reduction messages for HIV-positive and HIV-negative patients.	• The primary focus is similarly on emotional support for HIV-positive patients, but with stronger linkage to HIV care (*e.g*., the nurse does the CD4 blood test on the same day, and the patient is encouraged to attend follow-up sessions with the lay counsellor).
	• Lay counsellors are encouraged to provide up to three follow-up counselling sessions with HIV-positive patients.	• There is less focus on HIV-negative patients.

In both testing approaches there were no explicit steps outlined for ensuring effective linking of HIV positive patients to follow-up care, other than what the general clinical guidelines recommended. When patients tested HIV positive, they were given emotional support and encouraged to return for CD4 testing and a clinical examination. At the time, there was no standard protocol recommending CD4 testing on the same day. For the PITC approach, STI nurses offered HIV testing in the STI consultation and it was thought that this would be more advantageous for linking patients to care. For instance, the clinical consult allowed nurses to more easily request a CD4 test blood test on the same day and to encourage patients to return for a follow-up visit to receive their CD4 test result together with their other STI test results.

### Study population and sampling

The main controlled trial study was adequately powered to detect the hypothesised differences between HIV test uptake across arms, as described elsewhere [24]. The sample size for this nested follow-up study, however, was determined by the number of patients who tested HIV-positive in the controlled trial. As the sample size was pre-determined and we did not have any data to inform an estimation of an effect size, we did not perform a sample size calculation for this nested study. This is discussed as a limitation.

### Data collection

We started with a clinic-based HIV testing register with clinical and demographic data on all 930 patients who tested positive for HIV in the controlled trial between January and June 2007. In order to assess the linkage to care outcomes for these individuals, we then needed to match them to CD4 and viral load test records that are held in the National Health Laboratory System’s (NHLS) central database.

The first stage of the data collection process was to search for records of CD4 testing during a 12-month observation period from January to December 2007. We did not standardise the follow-up period due to logistical challenges. Data on CD4 lab records was not yet available beyond December 2007 to allow for standardised one year follow-up period for each patient (which would have required data to be available up to June 2008). We wanted to allow for the maximum follow-up period, rather than shortening the observation period to a standard 6 months. In effect, the observation period was between minimum 6 months and maximum of 12 months.

To match names with records, we searched the NHLS database using a range of patient identifiers, including name and surname, gender, and age as well as the patient’s clinic folder number. This process was necessary since clinic and laboratory records did not use unique patient identifiers (such as the unique South African citizen identity number or ID). We used an algorithm for the electronic search using multiple variables and possible variations of these variables (such as transposed patient name and surname). The algorithm also made use of a ‘sounds-like’ function to look for additional matches on names.

The results of the electronic matching process were then reviewed manually by one author (NL), duplicates and non-matches were removed, and a final set of true matches was identified, using the same range of variables mentioned above. The electronic and manual searches were done blind to trial allocation status. Quality control of the matching process was done in two steps. First we took a randomly selected sample of those HIV positive names for which no matching CD4 record could be found and we repeated the electronic search to determine if the initial search may have missed possible matches for CD4 records. Of the 20 randomly selected names, the second electronic search located a CD4 record for 1 name on the testing register. Given the time consuming process it required to perform the detailed individual search for matches for each of these 20 records, it was decided not to repeat the search for the full sample of patients with missing records. The outcome of this quality check (where 1 out of a sample of 20 was found to have been missed by the initial electronic search) was considered a reasonable underestimation (5%) by this search method, given the challenge presented by the absence of a unique patient identifier. The second step in the quality check related to the manual search and inclusion process and was aimed at ensuring the accuracy of the matching process for CD4 records. This involved an independent check of the manual record matches (by a senior colleague and co-author AB) of the accuracy of a random sample of 20 matched records. This independent quality check did not identify any discrepancies that could affect the main outcomes [26].

The second stage of the data collection process, done one year later, searched for viral load test records for the 622 HIV-positive patients who were identified in the first stage described above as having a CD4 test record. The same NHLS database was searched, this time for a 24-month observation period from January 2007 and December 2008. The observation periods were not the same for each patient, for similar reasons as described above for the CD4 testing record search. The minimum potential follow-up time therefore was 18 months and the maximum was 24 months. The search process was simplified using Link Plus software, a probabilistic record linkage programme initially designed by Centers for Disease Control and Prevention (CDC) (http://www.cdc.gov/cancer/npcr/tools/registryplus/lp.htm).

A proportion of patients (62 or 10%) who had CD4 test records after HIV testing, also had a record of CD4 testing *before* their HIV test date. The most likely explanation for this is that these patients knew their status to be HIV-positive and had previously undergone CD4 testing, but nevertheless re-tested for HIV during the intervention period. Such ‘known positive’ patients were excluded from the analysis of the proportion with viral load testing and from calculations of timing of CD4 and viral load testing. This is because there was a chance that their previous CD4 testing in another (or the same) service, may have been followed by a request for viral load testing to be done. The appearance of a record of viral load testing for these individuals after HIV testing and CD4 testing in the intervention period, therefore cannot automatically be ascribed to this intervention.

### Data analysis

Data analysis was performed using STATA statistical software, Version 10. Analysis compared the study arms with respect to the proportion of HIV-positive patients with CD4 records, their CD4 levels, and the proportion of ART-eligible patients with viral load records, as well as the timing of CD4 and viral load testing. Analysis used generalised linear models and took clustering by clinic into account using variance estimators in STATA which produced a robust Standard Error. Logistic regression was performed to analyse the difference between arms with respect to the primary and secondary outcomes. The Rank Sum and Weighted T-test was used for the cluster-level analysis. Kruskal-Wallis, a non-parametric, one-way analysis of variance test, was used to test for the equality of population median CD4 values. Kaplan-Meier estimates and Cox proportional hazards regressions were used for the time-to-event analysis of CD4 and viral load testing for those with observed events.

### Ethics

Ethical approval was obtained from the University of Cape Town Research Ethics Committee (HREC, REC#295/2007) and both the Cape Town and Western Cape Provincial health authorities gave permission for the evaluation.

## Results

### Participants

As reported elsewhere, the main outcome of the controlled trial was that the proportion of STI patients that tested for HIV was significantly higher in intervention than in the control arm (intervention = 1725 or 56.4% and control = 2821 or 42.6%, p = 0.037) [24]. As shown in Table 2, the proportion of patients who tested HIV-positive between study arms was comparable (326 or 18.6% in the intervention clinics and 604 or 21.4% in the control sites, p = 0.147), as was the median age (28 years in intervention and 26 years in control clinics). In both study arms, the majority (approximately two thirds) of the patients were female. The majority of females (91%) were 35 years or younger and the majority of males (83%) were 25 years and older.

**Table 2:** **Participants: proportion tested HIV positive and demographic information**

Study participants	Intervention % (n)	Control % (n)	P value
**1. STI patients tested for HIV**	56.4 (1752)	42.6 (2821)	0.037*
**2. Tested HIV-positive (as % of all tested)**	18.6 (326)	21.4 (604)	0.147
**3. Median age, years (range)**	28 (14–54)	26 (3–70)	0.162
**4. Gender: women**	62.9 (205)	66.7 (403)	0.423

*Significant difference at p < .05. Pearson chi^2,^.

The baseline comparison of sites in the main trial found no significant differences, except for one variable; the HIV acceptance rates of VCT patients (see Additional file 1). When adjusting for this difference in baseline characteristics, it did not change the interpretation of the findings in the main trial, as reported elsewhere [24].

### CD4 testing and viral load testing

The total number of potentially matching CD4 test records generated was 3 679 records. The manual search for matched CD4 records resulted in a final list of 824 true matches (narrowed down from the initial 3 679 potential matches). Some patients were found to have more than one CD4 test done. The first test record after HIV testing was included in the analysis and further CD4 records were removed from the dataset. After removal of these multiple CD4 records as well as erroneously duplicated records, the final sample of true matches for CD4 records was 622 HIV positive patients. The proportions of ‘known positives’ (those with CD4 test dates preceding their HIV test date in this study) were similar between arms with 11.4% (26 patients) in the intervention arm and 9.1% (36 patients) in the control arm (p = 0.363, Pearson chi^2^). As mentioned earlier, these ‘known positives’ were excluded from further analysis for viral load access and for the timing of access to CD4 and viral load testing.

Table 3 details the main outcomes on linkage to care for those who tested HIV-positive. In the pooled analysis of both arms, the proportion with a record of a CD4 testing was 66.9% or 622 of the 930 HIV-positive patients. Clinics with the PITC intervention did not have a higher proportion of patients with CD4 test records 69.9% or 228 patients in the intervention and 65.2% or 394 patients in the control sites, with an odds ratio of 0.82 (CI: 0.44-1.51; p = 0.526).

**Table 3:** **Linkage to care: CD4 and viral load testing done, median CD4 values and median time taken, by study arm**

Participants	Intervention	Control	Odds ratio	P value
**CD4 test done (as % all HIV-positive)**	69.9% (n = 228/326)	65.2% (n = 394/604)	Adjusted 0.82 (CI: 0.44-1.51)	0.526
			Unadjusted 0.81 (CI: 0.43-1.52)	0.504
**Viral load test done (as % of CD4 done)**	14.9% (n = 30/202)	10.9% (n = 39/358)	Adjusted 0.69 (CI: 0.42-1.12)	0.131
			Unadjusted 0.70 (CI: 0.48-1.02)	0.064
**Median CD4 (cells/mm** ^**3**^ **) (Range)**	386 (17–1509)	364 (11–1445)		0.446
**Median time (days) from HIV testing to CD4 testing (IQ range)**	3 (1–290)	2 (1–337)		0.646
**Median time (days) from CD4 to viral load testing for all CD4 categories (IQ range)**	245 (177–560)	306 (195–550)		0.622
**Median time (days) from CD4 to viral load testing for ART- eligible patients (CD4 ≤ 200 cells/mm** ^**3**^ **)** **(IQ range)**	214 (177–230)	288 (195–492)		0.007*

*Significant difference at p < .05. Pearson chi^2^, Log rank test.

Among the patients with CD4 test records, less than one quarter of patients were ART-eligible (according to CD4 level ≤200 cells/mm^3^), with 19% in the intervention and 21% in the control group being ART-eligible. When considering records of viral load testing for only the ART-eligible category (CD4 ≤ 200 cells/mm^3^), there was an absolute difference of 12.2% between arms in the proportion of patients who had a record of viral load test done, representing the largest difference across CD4 sub-groups (19 out of 39 or 33.3% intervention arm versus 16 out of 76 or 21.1% control arm). The numbers in these three CD4 sub-groups were, however, too small to test statistically for differences between arms.

In the pooled analysis (intervention and control groups), the proportion with viral load test records was 12.3% (irrespective of their CD4 counts and whether the test result was received). In the intervention arm the proportion was higher, but this difference was not found to be statistically significant (14.9% intervention versus 10.9% in the control arm); OR 0.69 (CI: 0.42-1.12; p = 0.131). Two tests were used in the cluster-level analysis and the p-values for the cluster-level analysis were not different from the p-values in the individual analysis (for CD4 test done, Rank sum p = 0.332 and Weighted t-test p = 0. 447 and for viral load test done Rank sum p = 0.101 and Weighted t-test p = 0.153).

### HIV immune status

The median CD4 levels of patients in both arms were similar (intervention = 386 cells/mm^3^, IQ range 17–1509 and control = 364 cells/mm^3^, IQ range 11–1445; p = 0.446, chi^2^ = 0.581). The study compared the proportions of patients with CD4 values in three categories from lowest to highest (≤200, 201–350 and >350 cells/mm^3^) and found no association between study arm and these CD4 categories. Using the highest CD4 category as a reference, there was no significant difference between arms for the lowest CD4 category (≤200 cells/mm^3^, p = 0.578) and for the middle CD4 category (201–350 cells/mm^3^, p = 0.672).

### Gaps in linkage to care

**Figure 1:**
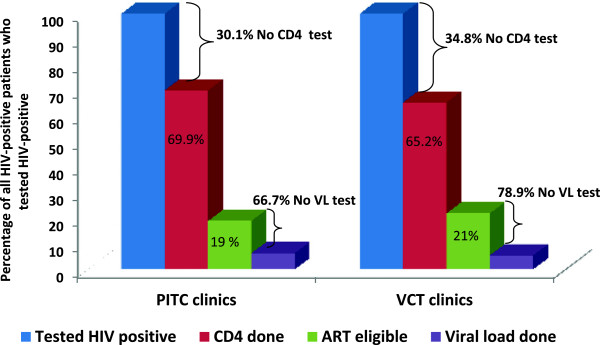
**Gaps in linkage to care: proportion of HIV-positive patients with no record of CD4 testing and the proportion of ART-eligible patients with no record of viral load testing, by study arm.** Figure 1 shows the main outcomes as a cascade of care to illustrate the gaps in linkage to care after testing HIV-positive in both arms of the study. The first gap in linkage to care was those HIV-positive patients who did not have a record of CD4 testing (indicated by the top set of brackets). The proportion of those patients with CD4 test records is represented by the second set of maroon-coloured bars. The third set of smaller green bars is the proportion of those with CD4 records who were ART-eligible. The last set of small, purple bars indicate those ART-eligible patients with records of viral load tests (expressed as a proportion of those who were ART-eligible). Finally, the second gap in linkage to care in the graph is the proportion of ART-eligible patients with no record of viral load testing (shown by the lower set of brackets).

In Figure 1, the main outcomes are presented as a cascade of care in both arms of the study. It shows the gap in linkage to CD4 testing and the gap in linkage to viral load testing for those who were ART-eligible (the latter being a proxy for the gap in linkage to ART). For those patients diagnosed HIV-positive, 30.1% in the intervention and 34.8% in the control arm, did not have a record of CD4 testing done - representing the first gap in linkage to care in the graph. The proportions of patients who were ART-eligible (shown in the smaller green bars), were 19% in the intervention group and 21% in the control group–this was illustrated as a proportion of those with CD4 test records (the second, maroon set of bars). The majority of ART-eligible patients did not have a record of a viral load test (66.7% for the intervention arm and 78.9% for the control arm) and this represents the second and larger gap in linkage to care.

### Time to accessing CD4 testing and viral load testing

The median time from HIV testing to accessing CD4 testing and from CD4 testing to viral load testing was similar across both arms, except for one sub-group (Table 3, final row). For the sub-group of ART-eligible patients, there was a significant difference between arms in time between their date of CD4 measurement and their date of viral load measurement (214 days in intervention vs. 288 days in control arm, HR: 0.417, 95% CI: 0.221-0.784, p = 0.007), indicating that those in the PITC intervention accessed viral load testing sooner.

## Discussion

The study found that in both arms, the majority (more than two thirds) of patients had a CD4 tests done. The PITC intervention did not improve the proportion of HIV positive patients with CD4 tests done, nor did it facilitate quicker access to CD4 testing. Most CD4 tests in both arms were done within less than a week of HIV testing and this, together with the relatively high level of CD4 tests done, point to a strength of the health service in terms of ensuring that appropriate blood tests are done as the first stage in linkage to care. However, this indicator cannot tell us what proportion of those with CD4 test records actually returned to the clinic and was informed of the test result.

PITC did not improve the proportion with viral load tests done (as a proportion of *all* those with CD4 tests done). Not all those with CD4 tests would have been eligible for initiation onto ART, so this indicator cannot strictly be taken as a measure of linkage to care in this study. The finding indicates that patients with higher CD4 counts are also accessing viral load tests (perhaps based on their clinical symptoms) and that even so, the proportions with access remained low. Due to small numbers, the study was not able to accurately detect differences between arms in proportions of ART-eligible patients with viral load tests.

The study did find that the time from CD4 testing to viral load testing for ART-eligible patients was approximately 2.5 months (74 days) shorter in the intervention arm as opposed to control arm. If viral load testing is used as a proxy for ART, then this indicates that ART-eligible patients may have been initiated onto ART more quickly in the clinics with the PITC intervention arm as compared to patients in clinics with the VCT approach to HIV testing. Given the small numbers observed, this finding needs to be confirmed by larger studies. The finding points to the potential benefit of the PITC intervention for those most in need of HIV follow-up care. The study was not able to investigate the possible reasons for this quicker access to viral load testing in the PITC intervention arm. One suggestion is that the closer involvement of nurses with the process of HIV testing within the clinical STI consultation, may have allowed nurses to more easily inform patients of the medical benefits of seeking immediate referral for ART initiation. Also, all STI patients are asked to return for a follow-up visit to receive their syphilis test result, so this could have provided patients with a ready-made opportunity to return to receive their CD4 test result and for timely referral for ART initiation.

The implication of removing the 10% ‘known positive’ patients from the analysis of viral load access and timing of CD4 and viral load testing is unclear. On the one hand, previous HIV testing and CD4 testing may imply greater contact with the health service and therefore increased chances for linkage to further care (e.g. viral load testing and ART initiation). On the other hand, the presence of multiple, parallel testing experiences may reflect on patient health seeking behaviour that might be limiting further linkage to care.

For the sample as a whole (across both arms), a higher proportion of patients who were ART-eligible (in the category CD4 ≤ 200 cells/mm^3^) had viral load tests done compared to those in higher CD4 categories. This could be taken as an indication that irrespective of the approach to HIV testing, those HIV-positive patients most in need of linkage to care were in fact prioritised by health providers for ART initiation, which is reassuring. The HIV immune status was similar across arms and in line with median CD4 levels reported in other LMIC and high-income countries [27–29] and this is perhaps indicative of the high risk status of STI patients in this setting [21, 30, 31].

The study identified gaps in linkage to care irrespective of the HIV testing approach used. Roughly one third of HIV-positive patients did not have a record of a CD4 test. Of greater concern is that more than two thirds of ART-eligible patients did not have a viral load test record, indicating that they may not have been initiated onto ART. This is in line with other studies pointing to a loss of linkage to care after HIV testing and prior to initiation of ART in South Africa and the sub-Saharan region [18–20]. A systematic review of linkage to care between testing and treatment in sub-Saharan Africa concluded that: “Studies of retention in pre-ART care report substantial loss of patients at every step, starting with patients who do not return for their initial CD4 count results and ending with those who do not initiate ART despite eligibility” [18].

Critics of expanded testing strategies such as PITC have expressed concern that whilst it may increase test uptake, it may also increase the number of HIV positive patients who are not effectively linked to care, especially in high prevalence and under-resourced settings such as South Africa. In this study, HIV positive STI patients in both arms had similar levels of linkage to care and similar gaps in linkage to care (except for the timing to viral load testing). One interpretation is that, at minimum, the PITC intervention did not disadvantage patients with respect to linkage to care and that it may hold opportunities for more timely linkage to care.

Nevertheless, the low levels of linkage to viral load testing for ART-eligible patients point to an extreme failure of the health system. If the push for scale-up of PITC is to reduce missed opportunities for diagnosis and linkage to care, then the still high loss to follow-up despite PITC is a major short coming of the initiative. A process evaluation of the main controlled trial on improving HIV test uptake, indicated that within a busy primary health care setting nurses were struggling to efficiently integrate HIV testing into the STI consultation, and that this may be the part of the reason for the smaller than anticipated effect size in HIV test uptake [32]. Similar limits on nurse time and challenges with efficient patient flow, may also account for the low linkage to care found in the PITC arm. Also, in this PITC intervention, nurses were referring their STI patients to the lay counsellor to receive the test result and post-test counselling (a mechanism introduced to save nurse time) and this break in continuity of care could have created missed opportunities for linking patients to care.

It could be argued that, even at the low levels of linkage to care found in this study, there may still be benefits arising from routinising HIV screening in this setting. Modelling of the effectiveness of routine annual HIV screening in South Africa concluded that it offered medical, prevention and cost-effectiveness benefits, even in highly constrained service delivery settings where the test acceptance rate and linkage to care rate could be as low as 20% [33]. Even so, efforts to improve linkage to care will become more critical given the increased demand on the health services from the change in ART-eligibility criteria (CD4 ≤ 350), and the increased numbers of people who have been testing since the introduction of the National Department of Health’s National HIV campaign that started in 2010 [34].

Linkage to and retention in HIV care has many complex components related to both patient and health systems factors - and the HIV testing approach is but one element of a set of moving parts that make up the health system delivering HIV linkage to care. New interventions such as PITC may be constrained by broader organisational barriers such as poor patient flow, poor monitoring and evaluation of patients and the absence of mechanisms for recalling HIV positive patients to ensure follow-up visits take place. This study did not investigate these and other components identified as requirements for successful linkage to care - such as for instance, strengthening service delivery and management capacity [18, 20, 35, 36].

New strategies to expand HIV testing approaches (as well as standard VCT approaches) could do more to include explicit steps aimed at increasing HIV prevention and linkage to care. When up-scaling PITC for STI patients (and for all patients in medical settings), the design of the intervention should allow for easy integration with the clinical consultation, without fragmenting the clinical tasks and the tasks related to HIV testing and linkage to care [32]. Also, it should be standard procedure for nurses and lay counsellors to request a CD4 test on the day of HIV testing and there should be a recall mechanism for patients who are ART-eligible based on their low CD4 count. Other factors found to promote linkage to care and that should be further investigated also for PITC interventions, include same day point-of- care CD4 testing, improved patient tracing and persistent reminders of follow-up visits, educational programmes to enhance staff friendliness and reducing the cost of accessing care [7, 18, 20, 35, 37].

The results of this study are likely to apply to sub-Saharan Africa, where health systems constraints may be prominent in limiting the potential impact of new interventions for expanding HIV testing and increasing linkage to care.

### Strengths and limitations of this study

The study has significant strengths and limitations. The strengths include the use of laboratory records which are a good proxy for follow-up care as they represent a centralised and comprehensive source of information about CD4 and viral load testing across all primary health facilities. It is also a more efficient and accurate way of extracting data on these follow-up clinical tests, rather than, for instance, reviewing individual patient folders that may be incomplete and harder to access. Another strength is that the laboratory record search was done province-wide and not limited to Cape Town, which increased the chances of detecting follow-up at clinics other than where the patient tested. The study also allowed for a reasonably long timeframe for follow-up periods, from a minimum of 6 months for CD4 testing and a minimum of 18 months for viral load testing.

A major limitation of the study was that the intervention and control sites were not randomly allocated which introduced the risk of bias. Baseline comparison of intervention and control sites for the main trial aimed to limit this bias to some extent. As reported earlier, none of the baseline variables were found to be significantly different, except one on ‘HIV test acceptance’ rates (see Additional file 1). We did not consider this a major risk for confounding because this variable referred to a different patient population (all clinic patients) and a different set of health providers (lay counsellors). In addition, while the difference in this VCT acceptance rate between the two arms was statistically significant, the rate itself was very high in both arms (93% and 85%), indicating a relatively high level of functioning in both arms [24].

The main controlled trial was not designed to measure access to care as a primary outcome and so the sample sizes did not allow for robust statistical analysis; the study was underpowered to detect smaller differences between the arms. Another limitation is the risk of underestimation of linkage to care when using laboratory records only; some patients may have started ART without a baseline viral load test being done. As mentioned earlier, during the course of this study, the health services removed the requirement for a baseline viral load test to be done at ART-initiation and this further complicates the interpretation of the findings on viral load testing.

Ideally, the data should have been double-extracted and double-entered for accuracy, which was not done. The absence of unique patient identifiers complicated the search for CD4 test records and may have resulted in underestimation of CD4 records. As described in the Methods section, we aimed to improve the accuracy of electronic and manual data extraction process. Searching only the NHSL database for the Western Cape Province only means that it would have missed patients who may have migrated to other provinces, especially to the neighbouring Eastern Cape Province. The effect of this on the results is likely to be small as migrations to the Eastern Cape are usually temporary transitions with patients tending to return to the Western Cape Province to seek out what is considered better medical care. The search for viral load testing was limited to only those who had CD4 test records and this would missed those patients who initiated ART without having a CD4 test done, based on their clinical symptoms only. This number is likely to be low for STI patients compared to other patient groups, like TB patients, where co-morbidity is likely to be accompanied by more serious and acute symptoms of illness. Although the study allowed for a reasonably long timeframe for follow-up periods, this may still be a limited time interval (as viral load testing may only have been done 9 months after a patient was initiated on ART). Nevertheless, the study design would have been strengthened if the follow-up period was standardised for all patients, even if this meant a shortened follow-observation period. The study also did not allow for assessing linkage to care for those not yet eligible for care or the rate of retention in care for ART patients. The effect of mortality on the study outcomes could not be determined as mortality is not tracked in routine health data or in laboratory records and some HIV positive patients with no follow-up data, may have died.

Further studies should consider both standardising and extending the follow-up period and should include investigation of follow up care for pre-ART patients (for example, monitoring 6 monthly follow-up CD4 testing for pre-ART patients). The introduction of the patients’ identity number (as a unique patient identifier) in routine records and/or for research purposes, could help to track mortality, as was done in a more recent trial on nurse-initiated ART [38].

Finally, the study was not able to ascertain what proportion of patients with a CD4 record actually returned to the clinic to get their CD4 test result, nor was it able to examine the reasons for the gaps in linkage to care, or to assess quality of care received. Larger studies using a longitudinal study design that can track patient care prospectively and/or retrospectively, as well as using multiple sources of data on care seeking (such as the newly introduced electronic patient record system for ART, in combination with laboratory data) could address some of the limitations identified in this study.

## Conclusion

Strategies for expanding HIV testing through routine HIV screening in medical settings should be evaluated for its effect on linkage to care after testing HIV positive as this can contribute to improved morbidity and mortality as well as reduced transmission of HIV. This study investigated linkage to care of STI patients following the introduction of PITC as compared to VCT for STI patients, and found no difference in the main outcomes of patients linked to CD4 testing and viral load testing. Numbers were too small to detect a difference in access to viral load testing for ART-eligible patients. In terms of secondary outcomes, ART-eligible patients had quicker access to viral load testing in the intervention arm – a finding that points to the potential benefit of having nurse clinicians offer HIV testing within a clinical consultation. This needs to be confirmed in larger studies. The study found major gaps linkage to care across both arms, especially for ART-eligible patients, where the majority were not linked to viral load testing and may therefore not have been initiated on ART, even though such a low CD4 count would have required urgent medical intervention. HIV testing strategies alone cannot address these gaps in linkage to care, (as this requires a range of health systems solutions), but it can do more to include explicit measures aimed at ensuring patients are linked to care shortly after testing HIV positive.

## Electronic supplementary material

Additional file 1: Table S1: Baseline comparison of intervention and control clinic demographics and service profile. Annual data for 2005. The table provides comparative baseline on a range of demographic and service outcome variables to investigate differences and similarities between the clinics in the intervention and control sites for the controlled trial that measured HIV test uptake [24]. The variables compared are total and STI caseload and performance outcome for the HIV testing services and the TB treatment services. There was no statistical differences between the two sites, except for one variable, that of ‘HIV test acceptance’ rate. (DOCX 13 KB)
