# Supplementary material for: A comparison of linkage to HIV care after provider-initiated HIV testing and counselling (PITC) versus voluntary HIV counselling and testing (VCT) for patients with sexually transmitted infections in Cape Town, South Africa
Source: BMC Health Serv Res. 2014 Aug 18;14:350. doi: 10.1186/1472-6963-14-350 (PMC4147183; doi:10.1186/1472-6963-14-350)
Supplement: Supplementary file 1 — Additional file 1: Table S1: Baseline comparison of intervention and control clinic demographics and service profile. Annual data for 2005. The table provides comparative baseline on a range of demographic and service outcome variables to investigate differences and similarities between the clinics in the intervention and control sites for the controlled trial that measured HIV test uptake [24]. The variables compared are total and STI caseload and performance outcome for the HIV testing services and the TB treatment services. There was no statistical differences between the two sites, except for one variable, that of ‘HIV test acceptance’ rate. (DOCX 13 KB) [file 12913_2013_3454_MOESM1_ESM.docx]

## Table S1: Baseline comparison of intervention and control clinic demographics and service profile. Annual data for 2005.

| Caseload | **Intervention clinics**  **(N=7)** | **Control clinics**  **(N=14)** | **P value** |
| --- | --- | --- | --- |
| 1. Total caseload: annual number of patients treated in 2005 | 504,679 | 822.395 | 0.40 |
| 1. Adult caseload: number of patients who were 5yrs and older | 334,758 | 600,142 | 0.66 |
| STI services |  |  |  |
| 1. STI-new: number of patients treated who presented with a new episode of STI | 8,466 | 12,377 | 0.26 |
| 1. STI load as a proportion of total adult caseload | 3% | 2% | 0.17 |
| HIV testing services |  |  |  |
| 1. VCT Total: number of patients who received voluntary counseling and testing (VCT) | 13,275 | 19,426 | 0.33 |
| 1. VCT load as a proportion of total adult caseload | 4% | 2% | 0.94 |
| 1. Proportion of VCT patients who were female | 56% | 54% | 0.33 |
| 1. HIV test acceptance: proportion of VCT patients who were tested for HIV | 93% | 85% | 0.03* |
| 1. HIV positive rate amongst patients who tested for HIV | 29% | 28% | 0.82 |
| 1. Lay Counsellor workload: the average number of VCT patients counseled per lay counsellor per day | 4.3 patients | 4.7 patients | 0.59 |
| TB treatment outcomes |  |  |  |
| 1. VCT for TB: proportion of New Smear Positive TB patients who received VCT | 79% | 77% | 0.63 |
| 1. TB success rate: proportion of New Smear Positive TB patients who were successfully treated (combined TB cure and TB completion rates) | 79% | 77% | 0.97 |

**Significance p> .05, Two sample t-test*

Data sources for variables numbered: 1-4=Routine Monthly Report (RMR); 5, 7, 8, 9, and 11=Voluntary Counselling and Testing quarterly reports; 10= Quarterly lay counsellor statistics report, 2005; 12= TB quarterly reports.
